# Dental Coverage and Care When Transitioning From Medicaid to Medicare

**DOI:** 10.1001/jamahealthforum.2024.4165

**Published:** 2024-11-22

**Authors:** Hawazin W. Elani, Benjamin D. Sommers, Dan Yuan, Ichiro Kawachi, Meredith B. Rosenthal, Renuka Tipirneni

**Affiliations:** 1 Department of Oral Health Policy and Epidemiology, Harvard School of Dental Medicine, Boston, Massachusetts; 2 Department of Health Policy and Management, Harvard T.H. Chan School of Public Health, Boston, Massachusetts; 3 Department of Medicine, Brigham & Women’s Hospital/Harvard Medical School, Boston, Massachusetts; 4 Institute for Quantitative Social Science, Harvard University, Boston, Massachusetts; 5 Department of Social and Behavioral Sciences, Harvard T.H. Chan School of Public Health, Boston, Massachusetts; 6 Divisions of General Medicine and Hospital Medicine, Department of Internal Medicine, University of Michigan, Ann Arbor

## Abstract

**Importance:**

Millions of adults with low incomes lose Medicaid eligibility when transitioning to Medicare at age 65 years. However, it remains unclear how this transition is associated with dental care.

**Objective:**

To examine the consequences of transitions from Medicaid to Medicare on coverage and use of dental services.

**Design, Setting, and Participants:**

Cross-sectional data from the Health and Retirement Study from 2014 to 2020 and a regression discontinuity design were used to compare changes in outcomes before and after turning age 65 years among a population likely to be Medicaid-eligible before age 65 years. The sample included adults aged 50 to 90 years who had not attended college in 28 states.

**Exposure:**

Transitions from Medicaid to Medicare at age 65 years.

**Main Outcomes and Measures:**

Health insurance (Medicaid, Medicare, dual coverage, private, and uninsurance), dental coverage (Medicaid, Medicare, private, or none), and having a dental visit and out-of-pocket dental spending during the previous 2 years.

**Results:**

Of the 15 837 study participants, 9510 (56.2% weighted) were female, 6984 (28.7% weighted) were Black individuals, Hispanic individuals, and individuals of other race (including American Indian, Alaskan Native, Asian, Native Hawaiian, and Pacific Islander individuals), and 8853 (71.3% weighted) were White; the mean (SD) age was 69.2 (10.3) years. Turning age 65 years was associated with an increase in Medicare coverage in states with Medicaid dental benefits (66.5 percentage points [pp]; 95% CI, 58.3-74.7) and those without dental benefits (67.8 pp; 95% CI, 52.6-83.0). There was a concurrent reduction in private coverage, Medicaid, and uninsured rates. For dental outcomes, in states providing Medicaid dental benefits, turning age 65 years was associated with a 13.1-pp decrease in the likelihood of dental coverage (95% CI, 10.7-15.5), largely due to the loss of Medicaid dental coverage. Among adults reporting being Black, Hispanic, or other race, there was a 3.9-pp decline in dental visits during the previous 2 years (95% CI, −6.1 to −1.7). In states without Medicaid dental benefits, turning age 65 years was associated with no change in the likelihood of dental coverage and a 15.6-pp increase in dental visits (95% CI, 6.3-25.0). Out-of-pocket dental spending decreased in both groups of states (−13.0% [95% CI, −24.2 to −0.1] and −19.2% [95% CI, −33.6 to −1.6], respectively).

**Conclusions and Relevance:**

The results of this cross-sectional study suggest that transitioning from Medicaid to Medicare at age 65 years was associated with a lower level of dental coverage and may increase barriers to accessing dental care for beneficiaries who had Medicaid dental coverage before age 65 years. However, for adults living in states without Medicaid dental benefits, the transition was associated with increased use of dental services and no change in overall dental coverage rates.

## Key Points

**Question:**

What is the association of transitioning from Medicaid to Medicare with coverage and use of dental services for adults with low incomes?

**Findings:**

In this cross-sectional study of 15 837 adults aged 50 to 90 years, when individuals in states with Medicaid dental benefits had access to dental coverage while younger than 65 years, the transition to Medicare was associated with worse rates of dental coverage and care. In contrast, individuals in states without dental benefits had less access to dental coverage when younger than 65 years, so the transition was associated with improved dental service use.

**Meaning:**

The study results suggest that current levels of dental care provision in Medicare are inadequate to eliminate gaps in coverage and access when adults transition from Medicaid to Medicare.

## Teaser

This cross-sectional study examines the consequences of transitions from Medicaid to Medicare on coverage and use of dental services.

## Introduction

The burden of dental disease is projected to rise substantially with the aging of the population.^1^ In turn, poor oral health has been associated with cognitive impairment and systemic conditions.^2,3^ Despite advances in other areas of health care, oral care remains a leading unmet health need in the US, particularly among marginalized populations,^4,5,6,7^ contributing to substantial disparities in oral health.^8,9,10^

The implementation of the Affordable Care Act (ACA) was associated with improved insurance coverage rates among adults with low incomes.^11^ However, adult dental care coverage in Medicaid varies widely among states, as it is not an essential health benefit for adults within the ACA. Prior studies found that the combination of Medicaid expansion and inclusion of Medicaid dental benefits was associated with improved coverage and access to dental care among adults with low incomes, with improved clinical indicators associated with oral health.^12,13,14,15^

Nevertheless, millions of adults with low incomes lose Medicaid eligibility when transitioning to Medicare at age 65 years. This is because Medicare eligibility is based on age, while Medicaid eligibility after age 65 years is determined by income (with more restrictive eligibility limits compared with Medicaid coverage for individuals younger than 65 years), assets, and disability status.^16^ Unlike Medicaid in many states, traditional Medicare does not provide dental coverage. Moreover, millions of working adults lose employer-based dental coverage at retirement. Medicare Advantage plans, which frequently include additional benefits, such as dental and vision care, are a major source of dental coverage for older adults.^17^ However, these plans can be associated with high out-of-pocket dental costs,^17^ creating challenges for those no longer in the labor force.^18^ Most recent estimates indicate that nearly 24 million Medicare beneficiaries have no dental coverage, and 47% did not visit the dentist during the previous year.^17^

It is important to understand the challenges that adults with low incomes face when transitioning from Medicaid to Medicare. The association of this transition with dental coverage, especially among racial and ethnic minority groups, as well as that with disparities in the use of dental services remain unclear. In this study, we leveraged the natural experiment created by transitioning from Medicaid to Medicare at age 65 years to examine the consequences on coverage and use of dental services. We stratified the analyses according to whether each state’s Medicaid program provides adult dental benefits.

## Methods

This cross-sectional study followed the Strengthening the Reporting of Observational Studies in Epidemiology (STROBE) reporting guidelines. The study was determined not to be human participants research by the institutional review board of Harvard University, which waived informed consent.

### Study Design and Population

We used a regression discontinuity (RD) design and leveraged the sharp discontinuity in Medicare eligibility at age 65 years to examine the association of transitions from Medicaid to Medicare with the study outcomes. We restricted the study sample to adults aged 50 to 90 years with 12 years of education or less (ie, no college education) to capture the population with a low socioeconomic status that would likely be eligible for Medicaid. While education is an imperfect proxy for income, the study design required us to identify those likely to be eligible for Medicaid but use a measure that is not substantially disrupted by turning age 65 years. Income fails this measure, since many adults retire at age 65 years, with substantial changes in their income biasing any comparison of adults older vs younger than 65 years.^19^ We also limited the study sample to adults residing in Medicaid expansion states after the 2014 Health and Retirement Study (HRS) survey wave (including those expanded in 2014 and 2015) to focus on the association of dental coverage benefits, rather than overall eligibility for Medicaid. We excluded nonexpansion states and those that expanded Medicaid after 2015.

We stratified the sample based on each state’s Medicaid adult dental benefits program. We defined a state as providing adult dental benefits in Medicaid if it offered more than emergency dental coverage to Medicaid beneficiaries.^20,21,22^ We excluded states that changed their dental benefits during the study period. The sample included 27 states and Washington, DC (eTable 1 in Supplement 1).

We also conducted separate analyses by race and ethnicity to capture the association of racism with health care access, as well as socioeconomic status, which is a determinant of Medicaid eligibility. Finally, we examined differences in dental coverage and service use between traditional Medicare and Medicare Advantage respondents aged 65 to 70 years. These results were descriptive, as we did not use the RD design to compare these 2 groups, because there is no comparable age group younger than 65 years stratified by traditional Medicare vs Medicare Advantage.

### Data Source

We analyzed data from the HRS, a nationally representative longitudinal survey of adults 50 years or older. The HRS collects data on key socioeconomic factors, labor force status, health conditions, health coverage, and health care use. HRS data are collected every 2 years.^23^ We obtained access to restricted state identifiers through the HRS and used the survey waves from 2014 to 2020.

### Study Variables

The study outcomes included 3 domains: medical coverage (Medicaid, Medicare, dual-eligible coverage, private, and uninsurance), dental coverage (Medicaid, Medicare, private, or none), and use of dental services. Respondents who reported having Medicaid and Medicare coverage were considered dual-eligible. Coverage status was based on the respondent’s coverage at the time of the interview.

Use of dental services outcomes included dental visits and out-of-pocket dental spending during the previous 2 years. Spending was measured in US dollars and expressed in real 2020 dollars for comparability across survey waves.^24^ All outcomes were self-reported and binary variables, except out-of-pocket dental spending, which was continuous.

We stratified the sample by self-reported race and ethnicity into non-Hispanic Black individuals, Hispanic individuals, non-Hispanic White individuals, and individuals of other races (including American Indian, Alaskan Native, Asian, Native Hawaiian, and Pacific Islander individuals). Because we lacked the statistical power, we combined the Black, Hispanic, and other race subpopulations.

### Statistical Analysis

To estimate the association between turning age 65 years and the study outcomes, we estimated the discontinuity using a local linear regression model, with a uniform kernel for each outcome. We used a data-driven approach to select the optimal bandwidth.^25^ Because the HRS is a longitudinal cohort with repeated observations over survey years, we estimated the models at the person-year level.^26^ We adjusted the models for sex, marital status, years of education, race and ethnicity, and state and year fixed effects. We used robust standard errors clustered by individuals to account for serial autocorrelation. For out-of-pocket dental spending, we first estimated the total annual out-of-pocket dental spending. We then used a 2-part model to account for the skewness of the data by applying the RD design separately in each part of the 2-part model.^27,28^ The first part of the model determined the probability of having any out-of-pocket dental spending, and the second part estimated the log-transformed out-of-pocket dental spending among those with spending.^29^

To examine differences in dental visits between traditional Medicare and Medicare Advantage, we used a multivariable logistic regression and generated predicted probabilities using marginal standardization. We also used multivariable linear regression and calculated marginal means for out-of-pocket dental spending. We adjusted models for sex, education, marital status, and state and year fixed effects. We used the HRS’s survey weights and Stata (StataCorp) for all analyses.^30^

### Additional Analyses

To test the assumptions of the study design and robustness of our findings, we first tested whether there was any evidence of manipulation of the assignment variable by examining the distribution of respondents around age 65 years, even though it is not possible to manipulate age for Medicare eligibility. Second, we conducted a falsification test by examining whether discontinuities in covariates trended smoothly at age 65 years.^31^ Third, we tested the robustness of the models to different bandwidths and triangular kernels. Fourth, we repeated the analyses by excluding covariates. Fifth, to ensure the robustness of the 2-part model, we estimated a single generalized linear model. Finally, we excluded individuals aged 65 and 66 years from the analyses of the outcomes of having seen a dentist and out-of-pocket dental spending. We used this donut approach because these questions had a 2-year look-back period, including periods before and after age 65 years.^32,33^

## Results

### Sample Characteristics

The study included 15 837 adults (weighted sample, 79 129 322 person-years) (Table 1). Of these, 1200 adults (weighted sample, 6 436 466 person-years) were in states without dental benefits, whereas 14 637 adults (weighted sample, 72 692 856 person-years) were in states that offer dental benefits. In both groups of states before age 65 years, most participants were White (63.3% and 67.1%, respectively) and female (52.1% and 61.8%, respectively).

**Table 1.** Characteristics of the Study Sample and Covariate Balance Greater and Less Than the Medicare Eligibility Threshold^a^

Characteristic	States without Medicaid dental benefits (unweighted, n = 1200 adults; weighted, n = 6 436 466 person-years)				States with Medicaid dental benefits (unweighted, n = 14 637 adults; weighted, n = 72 692 856 person-years)			
	Mean less than threshold^b^	Mean greater than threshold, pp^b^	Discontinuity greater vs less than threshold^c^		Mean less than threshold^b^	Mean greater than threshold, pp^b^	Discontinuity greater vs less than threshold^c^	
			(95% CI)	*P* value			(95% CI)	*P* value
Sex, %								
Female	61.8	63.3	−2.1 (−16.4 to 12.2)	.78	52.1	58.2	0.6 (−0.8 to 2.0)	.38
Male	38.2	36.7	2.1 (−12.2 to 16.4)	.78	48.0	41.8	−0.6 (−2.0 to 0.8)	.38
Race and ethnicity, %								
Black, Hispanic, or other race^d^	32.9	26.6	5.3 (−5.5 to 16.1)	.34	36.7	22.8	0.3 (−4.9 to 5.5)	.90
White	67.1	73.4	−5.3 (−16.1 to 5.5)	.34	63.3	77.2	−0.3 (−5.5 to 4.9)	.90
Married, %	55.6	51.0	1.8 (−5.0 to 8.7)	.60	61.2	53.0	−3.5 (−6.0 to −0.9)	.01

Abbreviation: pp, percentage points.

^a^ Data were from the Health and Retirement Study survey years 2014 to 2020. The study sample was limited to adults ages 50 to 90 years with up to 12 years of education in Affordable Care Act expansion states (28 states).

^b^ Mean older and younger than the age eligibility threshold for Medicare (age 65 years).

^c^ Discontinuities were estimated using a local linear regression and adjusted for age, state fixed effects, and year fixed effects. All analyses used robust standard errors clustered by individual.

^d^ Other race includes American Indian, Alaskan Native, Asian, Native Hawaiian, and Pacific Islander individuals.

### Changes in Outcomes Associated With Transitions From Medicaid to Medicare at Age 65 Years

**Figure 1.**
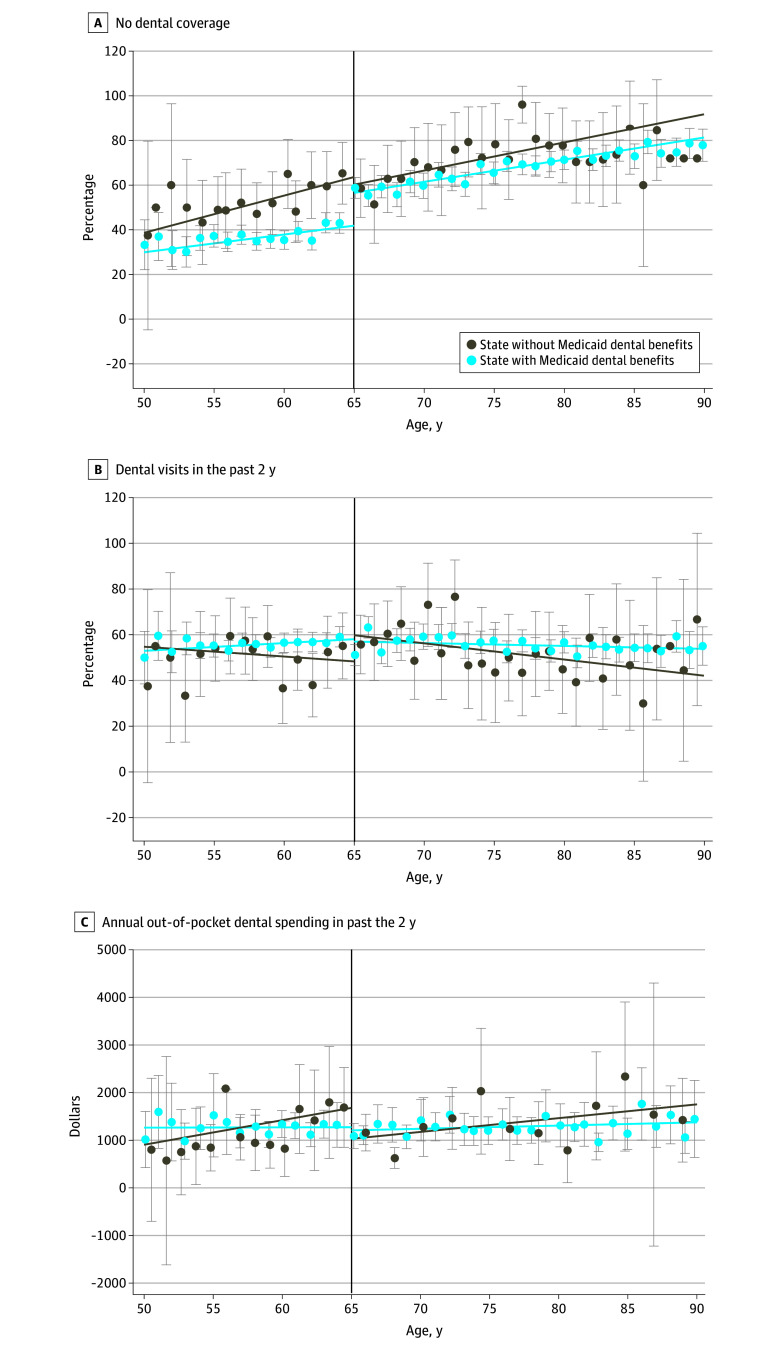
Dental Coverage, Access, and Out-of-Pocket Spending Greater and Less Than the Medicare Eligibility Threshold by States’ Dental Benefits in Medicaid Scatter plots of unadjusted proportion of outcomes greater and less than the Medicare eligibility threshold. Data are from the Health and Retirement Study (HRS) survey years 2014 to 2020. Study sample was limited to adults ages 50 to 90 years with up to 12 years of education in Affordable Care Act expansion states (28 states). All analyses are weighted by HRS survey weights and used robust standard errors clustered by individual. Whiskers indicate 95% CIs.

Turning age 65 years was associated with a marked increase in Medicare coverage in states with (66.5 percentage points [pp]; 95% CI, 58.3-74.7) and without Medicaid dental benefits (67.8 pp; 95% CI, 52.6-83.0) with concurrent reductions in private coverage, Medicaid, and uninsured rates (Table 2). Before age 65 years, individuals in states without Medicaid dental benefits were more likely to lack dental coverage than those in states providing dental benefits (52.1% vs 35.9%). However, turning age 65 years was associated with a 13.1-pp increase in the likelihood of no dental coverage (95% CI, 10.7-15.5) in states providing dental benefits, largely due to the loss of Medicaid dental coverage (−11.6 pp; 95% CI, −16.1 to −7.1) and a 4.5-pp increase in Medicare dental coverage (95% CI, 3.3-5.7). However, in states without Medicaid dental benefits, there were no changes in the likelihood of dental coverage (Table 2; Figure 1).

**Table 2.** Association of the Medicare Eligibility Threshold With Coverage, Access, and Out-of-Pocket Spending for Dental Services^a^

Characteristic	States without Medicaid dental benefits			States with Medicaid dental benefits		
	Mean younger than 65 years, %^b^	Adjusted discontinuity, pp (95% CI)^c^	*P* value	Mean younger than 65 years, %^b^	Adjusted discontinuity, pp (95% CI)^c^	*P* value
Medical coverage						
Medicaid	14.7	−17.6 (−25.2 to −10.0)	<.001	16.0	−10.7 (−14.5 to −6.9)	<.001
Medicare	9.3	67.8 (52.6 to 83.0)	<.001	10.5	66.5 (58.3 to 74.7)	<.001
Private	50.9	−28.5 (−51.8 to −5.2)	.02	55.9	−42.5 (−55.0 to −30.0)	<.001
Dual	4.8	2.4 (−7.7 to 12.6)	.64	5.6	6.9 (6.1 to 7.7)	<.001
Uninsured	22.3	−16.2 (−25.5 to −6.9)	<.001	14.2	−8.2 (−10.6 to −5.8)	<.001
Dental coverage						
Medicaid	1.6	−0.4 (−1.6 to 0.8)	.497	16.0	−11.6 (−16.1 to −7.1)	<.001
Medicare	2.2	5.1 (−5.6 to 15.7)	.35	2.0	4.5 (3.3 to 5.7)	<.001
Private	17.2	−1.7 (−6.0 to 2.6)	.43	20.2	−5.0 (−13.6 to 3.6)	.25
No dental coverage	52.1	−8.4 (−25.8 to 8.9)	.34	35.9	13.1 (10.7 to 15.5)	<.001
Dental visits during last 2 y	54.6	15.6 (6.3 to 25.0)	.001	60.0	−5.5 (−14.3 to 3.3)	.22
Annual out-of-pocket dental spending during last 2 y, $	2106.6	76.7(−471.1 to 624.5)	.78	1252.0	−293.9 (−899.5 to 311.6)	.34
Having any out-of-pocket dental spending	96.9	5.9 (−5.6 to 17.3)	.31	95.9	2.2 (1.7 to 2.8)	<.001
Annual out-of-pocket dental spending during the last 2 y among those with any spending, $^d^	2173.5	−19.2 (−33.6 to −1.6)	.03	1305.9	−13.0 (−24.2 to −0.1)	.048

Abbreviation: pp, percentage points.

^a^ Data were from the Health and Retirement Study survey years 2014 to 2020. The study sample was limited to adults ages 50 to 90 years with up to 12 years of education in Affordable Care Act expansion states (28 states). The full sample included 15 837 adults (state without dental benefits, n = 1200; state with dental benefits, n = 14 637).

^b^ Mean less than the age eligibility threshold for Medicare (age 65 years).

^c^ Adjusted discontinuities were estimated using a local linear regression with a uniform kernel. Models bandwidths ranged between 5.2 to 8.6. Models included individual-level covariates, state fixed effects, and year fixed effects. All analyses were weighted by Health and Retirement Study survey weights and used robust standard errors clustered by individual.

^d^ Out-of-pocket dental spending was log-transformed, and coefficients were converted to percentage changes.

For dental use outcomes (Table 2; Figure 1), in states without Medicaid dental benefits, turning age 65 years was associated with a 15.6-pp increase in the likelihood of any dental visits (95% CI, 6.3-25.0) and 19.2% reduction in out-of-pocket dental spending among those who had any dental expenses during the previous 2 years (95% CI, −33.6 to −1.6). In states with dental benefits, there was a 2.2-pp increase in the likelihood of any out-of-pocket dental spending (95% CI, 1.7-2.8) and 13.0% reduction in out-of-pocket dental spending (95% CI, −24.2 to −0.1) among those with any dental expenses during the previous 2 years.

### Association of Changes in Outcomes by Race and Ethnicity With Transitions From Medicaid to Medicare at Age 65 Years

**Figure 2.**
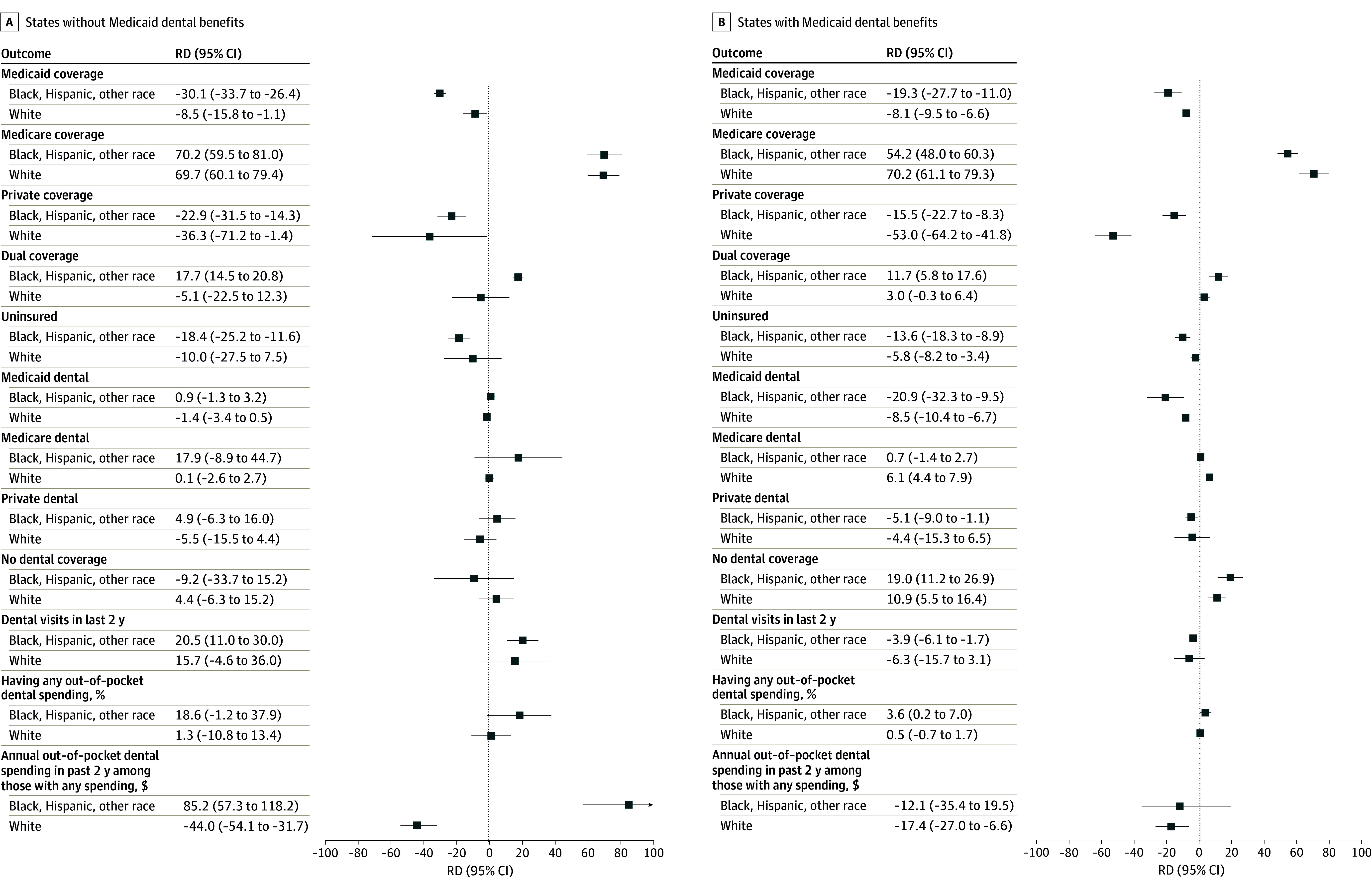
Association of the Medicare Eligibility Threshold With Coverage, Access, and Out-of-Pocket Spending for Dental Services by Race and Ethnicity Data are from the Health and Retirement Study (HRS) survey years 2014 to 2020. Study sample was limited to adults ages 50 to 90 years with up to 12 years of education in Affordable Care Act expansion states (28 states). Adjusted discontinuities are estimated using local linear regression with a uniform kernel. Bandwidths ranged between 5.2 to 8.6. Models included individual-level covariates, state fixed effects, and year fixed effects. All analyses are weighted by HRS survey weights and used robust standard errors clustered by individual. Other race includes American Indian, Alaskan Native, Asian, Native Hawaiian, and Pacific Islander individuals. Out-of-pocket dental spending was log-transformed, and coefficients were converted to percentage changes. The full sample included 15 837 adults (state without dental benefits, n = 1200; state with dental benefits, n = 14 637). RD indicates regression discontinuity.

Turning age 65 years was associated with increased Medicare coverage, reduced private and Medicaid coverage, and reduced uninsured rates for all racial and ethnic groups. This transition was more pronounced for Black individuals, Hispanic individuals, and individuals of other race than White individuals (Figure 2).

All racial and ethnic groups experienced an increase in the likelihood of no dental coverage when turning age 65 years in states with dental benefits. However, Black individuals, Hispanic individuals, and individuals of other race had a larger decrease in Medicaid dental coverage (−20.9 pp; 95% CI, −32.3 to −9.5) than White respondents (−8.5 pp; 95% CI, −10.4 to −6.7) and the likelihood of any dental coverage (19.0 pp [95% CI, 11.2-26.9] vs 10.9 pp [95% CI, 5.5-16.4], respectively). Among White respondents, there was a 6.1-pp increase in Medicare dental coverage (95% CI, 4.4-7.9). In states without dental benefits, turning age 65 years was not associated with any significant changes in dental coverage for all racial and ethnic groups.

Among Black individuals, Hispanic individuals, and individuals of other race in states without dental benefits, turning age 65 years was associated with a 20.5-pp (95% CI, 11.0-30.0) increase in dental visits during the previous 2 years. However, there was a reduction of 3.9 pp (95% CI, −6.1 to −1.7) for those residing in states providing dental benefits. These discontinuities were not significant among White respondents. Black individuals, Hispanic individuals, and individuals of other race experienced an increase in out-of-pocket dental spending among those with any spending in states without dental benefits. However, White respondents living in states without dental benefits experienced a larger decrease in spending than those in states with dental benefits.

### Differences in Dental Outcomes Between Traditional Medicare and Medicare Advantage After Age 64 Years

**Figure 3.**
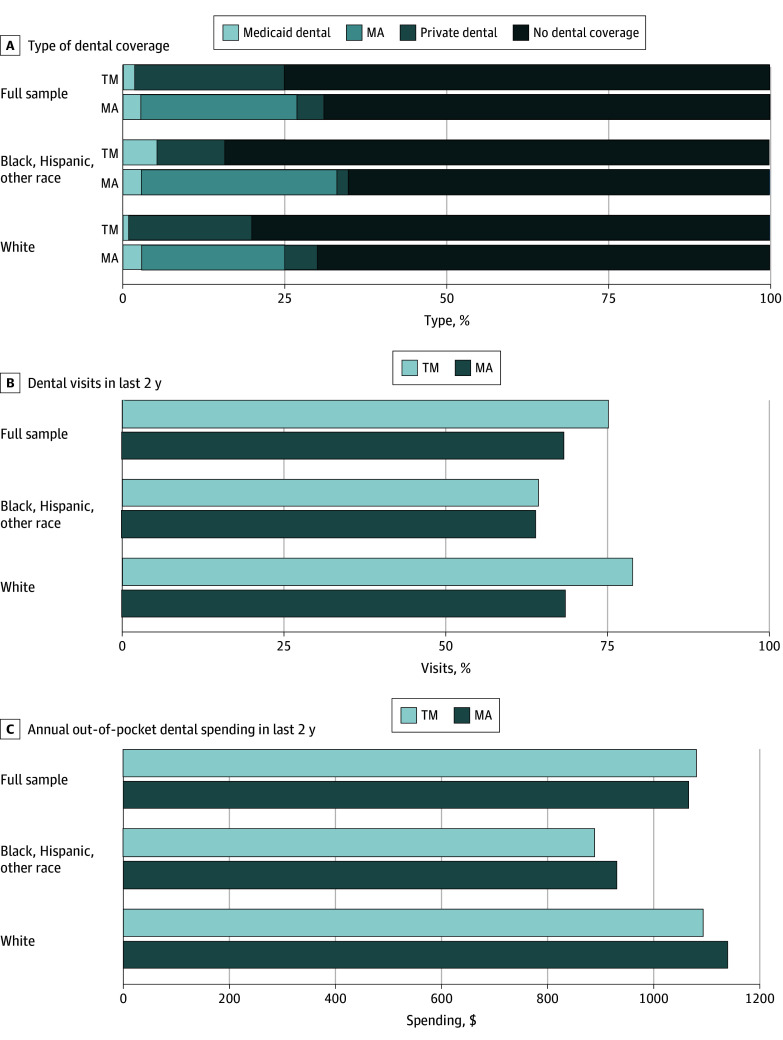
Dental Coverage, Access, and Out-of-Pocket Spending for Traditional Medicare (TM) vs Medicare Advantage (MA) Respondents by Race and Ethnicity Data are from the Health and Retirement Study (HRS) survey years 2014 to 2020. Study sample limited to adults ages 65 to 70 years with up to 12 years of education in Affordable Care Act expansion states (28 states). Other race includes American Indian, Alaskan Native, Asian, Native Hawaiian, and Pacific Islander individuals. Results were adjusted for individual-level covariates, state fixed effects, and year fixed effects. All analyses are weighted by HRS survey weights and used robust standard errors clustered by individual. Dental visits and annual out-of-pocket spending analyses were limited to respondents who reported having dental coverage.

We found that a higher percentage of traditional Medicare respondents lacked dental coverage (81.6%) than those with Medicare Advantage (69.2%) (Figure 3). In the full sample and among White respondents, Medicare Advantage respondents were less likely to have had a dental visit during the previous 2 years compared with traditional Medicare respondents (−7.0 pp [95% CI, −9.4 to −4.6] and −10.4 pp [95% CI, −14.4 to −6.4], respectively). However, there were no significant differences in dental visits for Black individuals, Hispanic individuals, and respondents of other races. We also found no significant difference in out-of-pocket dental spending in Medicare Advantage compared with traditional Medicare for the full sample or among racial and ethnic groups (eTable 2 in Supplement 1).

### Supplementary Analyses

The study findings were generally robust to different sensitivity checks. We did not find any significant discontinuities at age 65 years for demographic characteristics, including sex, race and ethnicity, and marital status, in states without dental benefits; married status was less common for those older than 65 years in states with dental benefits (Table 1; eFigures 1 and 2 in Supplement 1). Estimates without individual-level covariates, using different kernels and bandwidths, and from the generalized linear model were similar to our main analyses (eTables 3-5 and eFigures 3 and 4 in Supplement 1). When we excluded individuals aged 65 and 66 years in analyses of outcomes with a 2-year look-back period, changes remained largely similar to the main analysis (eTable 6 in Supplement 1).

## Discussion

The findings of this cross-sectional study suggest that turning age 65 years was associated with increased Medicare coverage and reduced private and Medicaid coverage in this sample of US adults with less than a college education. However, the association with dental coverage rates depended critically on the state’s Medicaid dental coverage policies. We found that in states that provided adult dental benefits in Medicaid, turning age 65 years was associated with significant dental coverage loss for all racial and ethnic groups and reduced use of dental services for Black individuals, Hispanic individuals, and individuals of other race. In contrast, for adults living in states without Medicaid dental benefits, the transition was associated with increased use of dental services and no significant changes in dental coverage rates. Adults in both groups of states experienced reductions in out-of-pocket dental spending at age 65 years.

These results underscore the importance of dental benefits in Medicaid and their association with disparities in dental coverage during the transition to Medicare. As expected, the loss of Medicaid dental coverage at age 65 years had no association with dental coverage in states that did not offer dental benefits. However, in states providing adult dental benefits under Medicaid, the transition was associated with a significant decrease in the likelihood of having any dental coverage, particularly for Black individuals, Hispanic individuals, and individuals of other races compared with White respondents. Additionally, the gains in Medicare dental coverage were observed only among White individuals. These findings suggest that people of racial and ethnic groups have greater difficulty maintaining coverage after turning age 65 years, raising concerns about exacerbating health disparities. Although people of racial and ethnic minority groups are more likely to enroll in Medicare Advantage plans,^34,35^ which often offer dental benefits, low-income populations may be less likely to take up Medicare dental coverage or use dental services due to higher cost-sharing in Medicare compared with Medicaid. Our analysis suggested that traditional Medicare respondents 65 years or older were more likely to be without dental coverage than those enrolled in Medicare Advantage. Efforts to support individuals approaching age 65 years in planning their transition to Medicare and understanding sources of dental coverage, particularly those enrolled in traditional Medicare, may reduce these losses and improve access to dental care.

Prior studies have shown that transitioning to Medicare after turning age 65 years is associated with increased access to health care and reduced out-of-pocket spending for previously uninsured adults^36,37^; our analysis suggests that this pattern of improved coverage and financial protection applies to dental coverage as well, but only for individuals in states without Medicaid dental coverage. This indicates that Medicare eligibility at age 65 years is associated with reduced financial barriers to accessing dental care, likely through alternative coverage, such as Medicare Advantage. In contrast, in states that provide Medicaid dental coverage, transitioning to Medicare at age 65 years appeared to be associated with reduced affordability of dental care and dental visits for Black individuals, Hispanic individuals, and individuals of other race.

Long-term exposure to dental benefits under Medicaid might be associated with individuals’ health care decisions regarding dental coverage as they age into Medicare. There was an increase in Medicare dental coverage only in states with Medicaid dental benefits, suggesting that individuals accustomed to having dental coverage under Medicaid may be more likely to seek ways to maintain it while transitioning to Medicare. Such preferences could be due to an increased awareness of the importance of dental care and an established routine of using dental services and may lead to individuals with Medicaid who are younger than 65 years to later enroll in Medicare Advantage plans that offer supplemental dental benefits instead of traditional Medicare, which lacks dental coverage. Alternatively, this pattern may also reflect differences in Medicare Advantage markets that are associated with the Medicaid state policy environment.

We found marked differences in dental expenditure between racial and ethnic groups. Underserved populations bear a disproportionate burden of dental disease^9,38,39,40^; therefore, they may need complex dental procedures, such as crowns and dentures, that are not covered by insurance. People of racial and ethnic minority groups are disproportionally enrolled in Medicare Advantage plans^34,35^; however, there are wide variations in cost-sharing across these plans,^17^ which can confuse beneficiaries, particularly those with low health literacy.^41,42^ As a result, people of racial and ethnic minority groups may enroll in lower-quality plans,^35^ with a low annual dollar cap and higher cost sharing, reducing access to dental care.^17^ In our analysis, we found no significant difference in out-of-pocket dental spending for respondents in Medicare Advantage compared with those in traditional Medicare.

### Limitations

This study aimed to examine adults with lower incomes who are likely eligible for Medicaid. Ideally, we would follow up individuals with Medicaid-range incomes from several years before to after turning age 65 years. However, since we could not follow up respondents for several years, we compared individuals younger than 65 years with individuals older than 65 years. Income changes significantly during this age range, so we were limited to using education as a proxy for potential Medicaid eligibility, which may have underestimated the association of the transition. Second, we lacked measures of clinical need to assess needed dental care, as well as data on rurality, which is associated with limited clinician access and transportation challenges. Third, our aim was to examine how state Medicaid dental policy affects individuals transitioning to Medicare at age 65 years. Examining the additional association of traditional Medicare and Medicare Advantage requires a different sampling and analytic approach, because the population younger than 65 years cannot be readily divided into 2 appropriate comparison groups for an RD design. Finally, we only examined states with Medicaid expansion as of 2015 that did not change their dental coverage policies. Additionally, the sample size for states without dental coverage was small. Therefore, our findings may not be generalizable to all states.

## Conclusions

The findings of this cross-sectional study suggest that transitioning from Medicaid to Medicare at age 65 years is associated with increased barriers to accessing dental care for beneficiaries in states providing Medicaid dental coverage before age 65 years. This highlights the inadequacy of current Medicare dental provisions, primarily in Medicare Advantage, in closing these gaps. The results support federal initiatives to cover supplemental benefits, including dental benefits, in Medicare, as well as efforts to improve data collection to evaluate Medicare Advantage dental coverage quality.^43,44^ Additionally, the inclusion of dental care for adults as an essential health benefit may expand dental coverage in the Marketplace, improving dental health for privately insured individuals before age 65 years.^45^ At the state level, maintaining and expanding Medicaid adult dental benefits may be associated with long-term cost savings in Medicare by ensuring better oral health during the transition. These findings underscore the need for coordinated state and federal efforts to provide continuous dental coverage and support strategies to mitigate the adverse association of this transition.

## Notes

Supplement 1. eTable 1. States classification based on Medicaid adult dental benefits status during the study period eTable 2. Adjusted differences in access, and out-of-pocket spending for Traditional Medicare versus Medicare Advantage respondents by race and ethnicity eTable 3. Sensitivity analyses excluding individual-level covariates eTable 4. Sensitivity analyses excluding individual-level covariates by race and ethnicity eTable 5. Sensitivity analyses for out-of-pocket dental spending using generalized linear model eTable 6. Sensitivity analyses excluding individual aged 65 and 66 for outcomes with 2-year look-back period eFigure 1. Distribution of the study sample around the Medicare age eligibility threshold eFigure 2. Marital status above and below the Medicare eligibility threshold by states dental benefits in Medicaid eFigure 3. Sensitivity of regression discontinuity estimates to alternative bandwidth and kernel for medical coverage outcomes eFigure 4. Sensitivity of regression discontinuity estimates to alternative bandwidth and kernel for dental coverage, access, and out-of-pocket spending

Supplement 2. Data sharing statement
