# Error in Introduction and Figure 1

**DOI:** 10.1001/jamahealthforum.2024.5090

**Published:** 2024-12-20

The Original Investigation “Dental Coverage and Care When Transitioning From Medicaid to Medicare,”^1^ published November 22, 2024, was updated to reflect more accurate language in the Introduction regarding estimates of Medicare beneficiaries without dental coverage and who did not visit the dentist in the previous year, as well as to change the color of the 95% CI whiskers in Figure 1 to improve clarity. This article was corrected online.
