# Supplementary material for: Dental Coverage and Care When Transitioning From Medicaid to Medicare
Source: JAMA Health Forum. 2024 Nov 22;5(11):e244165. doi: 10.1001/jamahealthforum.2024.4165 (PMC11584926; doi:10.1001/jamahealthforum.2024.4165)
Supplement: Supplement 1. — eTable 1. States classification based on Medicaid adult dental benefits status during the study period eTable 2. Adjusted differences in access, and out-of-pocket spending for Traditional Medicare versus Medicare Advantage respondents by race and ethnicity eTable 3. Sensitivity analyses excluding individual-level covariates eTable 4. Sensitivity analyses excluding individual-level covariates by race and ethnicity eTable 5. Sensitivity analyses for out-of-pocket dental spending using generalized linear model eTable 6. Sensitivity analyses excluding individual aged 65 and 66 for outcomes with 2-year look-back period eFigure 1. Distribution of the study sample around the Medicare age eligibility threshold eFigure 2. Marital status above and below the Medicare eligibility threshold by states dental benefits in Medicaid eFigure 3. Sensitivity of regression discontinuity estimates to alternative bandwidth and kernel for medical coverage outcomes eFigure 4. Sensitivity of regression discontinuity estimates to alternative bandwidth and kernel for dental coverage, access, and out-of-pocket spending [file jamahealthforum-e244165-s001.pdf]

Supplemental Online Content

Elani HW, Sommers BD, Yuan D, Kawachi I, Rosenthal MB, Tipirneni R. Dental coverage and care when transitioning from Medicaid to Medicare. *JAMA Health Forum*. 2024;5(11):e244165. doi:10.1001/jamahealthforum.2024.4165

eTable 1. States classification based on Medicaid adult dental benefits status during the study period
eTable 2. Adjusted differences in access, and out-of-pocket spending for Traditional Medicare versus Medicare Advantage respondents by race and ethnicity
eTable 3. Sensitivity analyses excluding individual-level covariates
eTable 4. Sensitivity analyses excluding individual-level covariates by race and ethnicity
eTable 5. Sensitivity analyses for out-of-pocket dental spending using generalized linear model
eTable 6. Sensitivity analyses excluding individual aged 65 and 66 for outcomes with 2-year look-back period
eFigure 1. Distribution of the study sample around the Medicare age eligibility threshold
eFigure 2. Marital status above and below the Medicare eligibility threshold by states dental benefits in Medicaid
eFigure 3. Sensitivity of regression discontinuity estimates to alternative bandwidth and kernel for medical coverage outcomes
eFigure 4. Sensitivity of regression discontinuity estimates to alternative bandwidth and kernel for dental coverage, access, and out-of-pocket spending

This supplemental material has been provided by the authors to give readers additional information about their work.

© 2024 Elani HW et al. JAMA Health Forum.

eTable 1. States classification based on Medicaid adult dental benefits status during the study period

Expansion states	
**States without Medicaid dental benefits**	**States with Medicaid dental benefits**
AZ	AK
DE	AR
HI	CA
MD	CO
NH	CT
NV	DC
WV	IA
	IL
	IN
	KY
	MA
	MI
	MN
	ND
	NJ
	NM
	NY
	OH
	OR
	PA
	RI
	VT
	WA

Note. Study sample included 28 states. Not all states listed in the table are included in the analysis. Due to HRS disclosure requirement and data use agreement we are unable to list the names of individual states in the study sample. Definitions of Medicaid coverage categories for adult dental benefits according to the Center for Health Care Strategies are: "*Emergency Only*: Relief of pain under defined emergency situations. *Limited*: Fewer than 100 diagnostic, preventive, and minor restorative procedures recognized by the American Dental Association; per-person annual expenditure for care is $1,000 or less. *Extensive*: A comprehensive mix of services, including more than 100 diagnostic, preventive, and minor and major restorative procedures approved by the ADA; per-person annual expenditure cap is at least $1,000". [24] DE and WV added dental benefits in 2021 and HI dded dental benefits in 2020.

© 2024 Elani HW et al. JAMA Health Forum.

eTable 2. Adjusted differences in access, and out-of-pocket spending for Traditional Medicare versus Medicare Advantage respondents by race and ethnicity

	Traditional Medicare	Medicare Advantage	Medicare Advantage vs Traditional Medicare	
			Difference (95% CI)	*P* Value
**Dental visits in last 2 years, %** [a]				
Full sample	75.6	68.7	-7.0 (-9.4, -4.6) [c]	<.001
Black, Hispanic, other race	64.7	64.3	-0.3 (-4.7, 4.0) [c]	0.878
White	79.3	68.9	-10.4 (-14.4, -6.4) [c]	<.001
**Annual out-of-pocket dental spending in last 2 years, dollars** [b]				
Full sample	$1,082.0	$1,066.8	-15.2 (-448.7, 418.2)	0.934
Black, Hispanic, other race	$889.8	$932.6	42.8 (-186.9, 272.5)	0.652
White	$1,094.5	$1,141.9	47.4 (-339.3, 434.0)	0.774

Note. Data are from the Health and Retirement Study (HRS) survey years 2014 to 2020. Study sample limited to adults ages 65 to 70 years with up to 12 years of education in ACA expansion states (28 states) who reported having dental coverage. Other race includes American Indian, Alaskan Native, Asian, Native Hawaiian, and Pacific Islander. Results from survey-weighted [a]logistic and [b]linear regression adjusted for individual-level covariates, state fixed effects, and year fixed effects. [c]Odds ratios were converted to predicted probabilities using marginal standardization for ease of interpretability. All analyses are weighted by HRS survey weights and used robust standard errors clustered by individual.

© 2024 Elani HW et al. JAMA Health Forum.

eTable 3. Sensitivity analyses excluding individual-level covariates

	States without Medicaid dental benefits			States with Medicaid dental benefits		
	Mean below 65 [a]	Adjusted discontinuity pp (95% CI) [b]	*P* value	Mean below 65 [a]	Adjusted discontinuity pp (95% CI) [b]	*P* value
**Medical coverage**						
Medicaid coverage, %	14.7	-17.1 (-22.6, -11.6)	<.001	16.0	-11.8 (-14.5, -9.2)	<.001
Medicare coverage, %	9.3	68.6 (56.5, 80.7)	<.001	10.5	67.0 (59.3, 74.7)	<.001
Private coverage, %	50.9	-28.9 (-50.7, -7.0)	0.010	55.9	-43.3 (-54.0, -32.6)	<.001
Dual coverage, %	4.8	3.4 (-6.8, 13.6)	0.514	5.6	7.3 (6.3, 8.2)	<.001
Uninsured, %	22.3	-17.6 (-26.8, -8.4)	<.001	14.2	-7.9 (-10.2, -5.5)	<.001
**Dental coverage**						
Medicaid dental, %	1.6	-0.5 (-1.8, 0.7)	0.422	16.0	-11.9 (-15.4, -8.4)	<.001
Medicare dental, %	2.2	6.0 (-4.8, 16.9)	0.277	2.0	4.6 (3.3, 5.9)	<.001
Private dental, %	17.2	-1.2 (-5.7, 3.2)	0.590	20.2	-5.1 (-13.5, 3.2)	0.229
No dental coverage, %	52.1	-9.5 (-27.5, 8.5)	0.302	35.9	13.4 (10.6, 16.2)	<.001
**Dental use**						
Dental visits in last 2 years, %	54.6	14.3 (3.3,25.3)	0.011	60.0	-6.3 (-15.2, 2.5)	0.161
Annual out-of-pocket dental spending in last 2 years, dollars	$2,106.6	162.3 (-21.7, 346.3)	0.084	$1,252.0	-270.8 (-847.3, 305.7)	0.357
Having any out-of-pocket dental spending (%)	96.9	5.6 (-5.1,16.3)	0.308	95.9	2.2 (1.6, 2.9)	<.001
Annual out-of-pocket dental spending in last 2 years among those with any spending, dollars [c]	$2,173.5	-13.4 (-21.4, -4.6)	0.004	$1,305.9	-11.9 (-22.1, -0.4)	0.043

Note. Data are from the Health and Retirement Study (HRS) survey years 2014 to 2020. Study sample limited to adults ages 50 to 90 years with up to 12 years of education in ACA expansion states (28 states). pp=percentage points. [a] Mean below the age eligibility threshold for Medicare (age 65). [b] Adjusted discontinuities are estimated using local linear regression with a uniform kernel. Models included age, state fixed effects, and year fixed effects. All analyses are weighted by HRS survey weights and used robust standard errors clustered by individual. [c]Out-of-pocket dental spending was log-transformed, and coefficients were converted to percentage changes. The full sample included 15,837 adults (state without dental benefits=1,200, state with dental benefits=14,637).

© 2024 Elani HW et al. JAMA Health Forum.

eTable 4. Sensitivity analyses excluding individual-level covariates by race and ethnicity

	States without Medicaid dental benefits			States with Medicaid dental benefits		
	Mean below age 65[a]	Adjusted discontinuity pp (95% CI)[b]	P Value	Mean below age 65[a]	Adjusted discontinuity pp (95% CI)[b]	P Value
**Medical coverage**						
**Medicaid coverage**						
Black, Hispanic, other race	20.8	-29.9 (-32.4, -27.5)	<.001	27.0	-21.7(-25.5, -18.0)	<.001
White	11.7	-9.4 (-17.1, -1.8)	0.015	10.0	-8.1 (-9.5, -6.6)	<.001
**Medicare coverage**						
Black, Hispanic, other race	5.9	72.1 (64.7, 79.6)	<.001	9.7	55.7(49.2, 62.2)	<.001
White	11.0	69.7 (60.3, 79.2)	<.001	10.9	70.1(61.3, 79.0)	<.001
**Private coverage**						
Black, Hispanic, other race	46.8	-23.1 (-25.4, -20.8)	<.001	37.0	-17.9(-23.8, -12.0)	<.001
White	52.9	-32.5 (-73.6, 8.6)	0.121	66.8	-53.4(-64.3, -42.6)	<.001
**Dual coverage**						
Black, Hispanic, other race	4.3	16.9 (11.2, 22.7)	<.001	9.1	13.8 (9.2, 18.4)	<.001
White	5.1	-6.0 (-24.2, 12.2)	0.518	3.6	3.3 (0.3, 6.4)	0.034
**Uninsured**						
Black, Hispanic, other race	24.3	-20.9 (-23.2, -18.7)	<.001	21.8	-12.0 (-15.5, -8.6)	<.001
White	21.3	-9.6 (-30.4, 11.1)	0.362	9.9	-5.8 (-8.2, -3.5)	<.001
**Dental coverage**						
**Medicaid dental**						
Black, Hispanic, other race	0.6	1.0 (-1.4, 3.4)	0.411	25.3	-21.3 (-31.5, -11.2)	<.001
White	2.1	-1.5 (-3.9, 1.0)	0.243	10.7	-8.4 (-10.6, -6.3)	<.001
**Medicare dental**						
Black, Hispanic, other race	2.5	18.2 (-12.6, 49.0)	0.246	3.2	1.0 (-0.9, 2.9)	0.298
White	2.1	-0.1 (-2.7, 2.7)	0.995	1.3	6.1 (4.2, 8.0)	<.001
**Private dental**						
Black, Hispanic, other race	14.9	5.6 (-4.1, 15.3)	0.255	14.5	-5.0 (-9.0, -1.0)	0.013
White	18.3	-5.4 (-15.8, 5.0)	0.306	23.5	-4.7 (-15.5, 6.2)	0.399
**No dental coverage**						
Black, Hispanic, other race	66.1	-12.3 (-36.2, 11.6)	0.314	40.4	19.3 (14.1, 24.6)	<.001
White	45.5	0.4 (-9.7, 10.6)	0.933	33.4	10.9 (5.4, 16.5)	<.001
**Dental use**						
**Dental visits in the last 2 years**						
Black, Hispanic, other race	52.4	20.3 (6.9, 33.7)	0.003	53.9	-5.6 (-8.0, -3.1)	<.001
White	55.8	20.2 (-8.9, 49.1)	0.172	63.6	-6.7 (-16.0, 2.7)	0.163
**Annual out-of-pocket dental spending in last 2 years, dollars**						
Black, Hispanic, other race	$755.3	463.0 (270.2, 655.8)	<.001	$1,090.9	-16.8 (-211.8, 178.2)	0.866
White	$2,841.8	-451.3 (-1434.6,531.9)	0.368	$1,312.4	-344.8 (-1,066.2,376.5)	0.349
**Having any out-of-pocket dental spending (%)**						
Black, Hispanic, other race	97.9	18.1 (0.5, 35.7)	0.043	93.9	3.2 (-0.1, 6.5)	0.059
White	96.4	0.6 (-10.7, 11.8)	0.920	96.6	0.3 (-0.9, 1.5)	0.667
**Annual out-of-pocket dental spending in last 2 years among those with any spending, dollars**[c]						
Black, Hispanic, other race	$771.6	121.1 (23.4, 296.1)	0.008	$1,161.8	-11.8 (-33.3, 16.7)	0.380
White	$2,948.1	-45.0 (-53.3,-35.1)	<.001	$1,358.4	-16.2 (-26.9, -3.9)	0.011

Note. Data are from the Health and Retirement Study (HRS) survey years 2014 to 2020. Study sample limited to adults ages 50 to 90 years with up to 12 years of education in ACA expansion states (28 states). pp=percentage points. [a] Mean below the age eligibility threshold for Medicare (age 65). [b] Adjusted discontinuities are estimated using local linear regression with a uniform kernel. Models included age, state fixed effects, and year fixed effects. All analyses are weighted by HRS survey weights and used robust standard errors clustered by individual. Other race includes American Indian, Alaskan Native, Asian, Native Hawaiian, and Pacific Islander. [c]Out-of-pocket dental spending was log-transformed, and coefficients were converted to percentage changes. The full sample included 15,837 adults (state without dental benefits=1,200, state with dental benefits=14,637).

© 2024 Elani HW et al. JAMA Health Forum.

eTable 5. Sensitivity analyses for out-of-pocket dental spending using generalized linear model

	States without Medicaid dental benefits			States with Medicaid dental benefits		
	Mean below 65 [a]	Adjusted discontinuity pp (95% CI) [b]	*P* value	Mean below 65 [a]	Adjusted discontinuity pp (95% CI) [b]	*P* value
**Annual out-of-pocket dental spending in last 2 years, dollars**						
Full sample	$2106.6	-39.0 (-68.8, -9.1)	0.010	$1,252.0	-40.6 (-62.0, -19.2)	<.001
Black, Hispanic, other race	$755.3	14.6 (-23.2, 52.4)	0.448	$1,090.9	-29.8 (-56.2, -3.4)	0.027
White	$2841.8	-19.0 (-45.7, 7.7)	0.163	$1,312.4	-46.0 (-60.9, -31.0)	<.001

Note. Data are from the Health and Retirement Study (HRS) survey years 2014 to 2020. Study sample limited to adults ages 50 to 90 years with up to 12 years of education in ACA expansion states (28 states). pp=percentage points. Other race includes American Indian, Alaskan Native, Asian, Native Hawaiian, and Pacific Islander. [a] Mean below the age eligibility threshold for Medicare (age 65). [b] Adjusted discontinuities are estimated using generalized linear model with a log function. Models included individual-level covariates, state fixed effects, and year fixed effects. All analyses are weighted by HRS survey weights and used robust standard errors clustered by individual.

© 2024 Elani HW et al. JAMA Health Forum.

eTable 6. Sensitivity analyses excluding individual aged 65 and 66 for outcomes with a 2-year look-back period

	States without Medicaid dental benefits			States with Medicaid dental benefits		
	Mean below 65 [a]	Adjusted discontinuity pp (95% CI) [b]	*P* value	Mean below 65 [a]	Adjusted discontinuity pp (95% CI) [b]	*P* value
**Dental visits in last 2 years, %**						
Full sample	54.7	35.4 (18.2, 52.6)	<.001	60.0	-10.2 (-19.9, -0.4)	0.041
Black, Hispanic, other race	52.4	46.5 (30.3, 62.7)	<.001	53.9	-11.1 (-16.3, -5.9)	<.001
White	55.8	25.9 (6.9, 45.0)	0.008	63.6	-4.1 (-14.8, 6.7)	0.458
**Annual out-of-pocket dental spending in last 2 years, dollars**						
Full sample	$2106.6	19.4 (-585.0, 623.9)	0.950	$1,252.0	-236.8 (-940.6, 467.1)	0.510
Black, Hispanic, other race	$755.3	91.2 (-15.5, 197.9)	0.094	$1,090.9	7.6 (-243.4, 258.7)	0.953
White	$2841.8	-18.1 (-979.4, 943.2)	0.971	$1,312.4	-305.3 (-1,125.6, 515.0)	0.466
**Having any out-of-pocket dental spending (%)**						
Full sample	96.9	-0.6 (-12.5, 11.3)	0.921	95.9	1.9 (-0.5, 4.2)	0.122
Black, Hispanic, other race	97.9	30.1 (-5.0, 65.2)	0.093	93.9	8.3 (3.7, 12.9)	<.001
White	96.4	-7.0 (-16.9,2.9)	0.168	96.6	0.6 (-2.2, 3.4)	0.663
**Annual out-of-pocket dental spending in last 2 years among those with any spending, dollars** [c]						
Full sample	$2173.5	-28.4 (-55.0,14.0)	0.159	$1,305.9	-18.4 (-35.5, 3.4)	0.092
Black, Hispanic, other race	$771.6	39.3 (4.1,86.3)	0.026	$1,358.4	-4.8 (-42.8, 58.4)	0.851
White	$2948.1	-47.6 (-57.5,-35.3)	<.001	$1,161.8	-20.9 (-39.4, 3.4)	0.086

Note. Data are from the Health and Retirement Study (HRS) survey years 2014 to 2020. Study sample limited to adults ages 50 to 90 years with up to 12 years of education in ACA expansion states (28 states). pp=percentage points. Other race includes American Indian, Alaskan Native, Asian, Native Hawaiian, and Pacific Islander. [a] Mean below the age eligibility threshold for Medicare (age 65). [b] Adjusted discontinuities are estimated using local linear regression with a uniform kernel. Models included age, state fixed effects, and year fixed effects. All analyses are weighted by HRS survey weights and used robust standard errors clustered by individual.[c]Out-of-pocket dental spending was log-transformed, and coefficients were converted to percentage changes. We used this “donut” regression discontinuity approach to exclude individuals aged 65 and 66 because these questions had a 2-year look-back period which can include responses at age 65.

© 2024 Elani HW et al. JAMA Health Forum.

eFigure 1. Distribution of the study sample around the Medicare age eligibility threshold

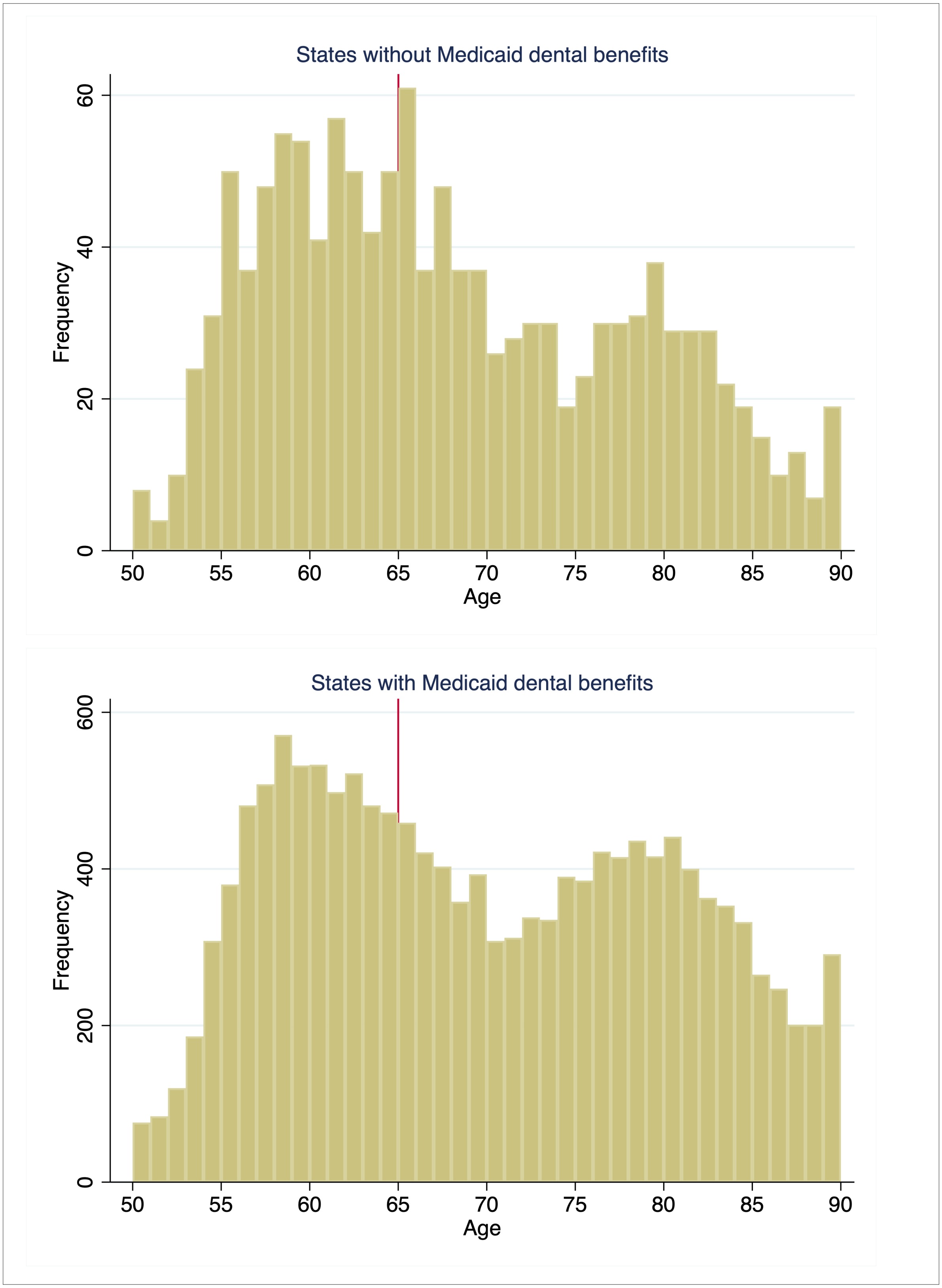

Note. Kernel density plot overlaid on frequency histogram (n=40 bins). Data are from the Health and Retirement Study (HRS) survey years 2014 to 2020. Study sample limited to adults ages 50 to 90 years with up to 12 years of education in ACA expansion states (28 states). State without dental benefits included 1,200 adults and state with dental benefits included 14,637.

© 2024 Elani HW et al. JAMA Health Forum.

eFigure 2. Marital status above and below the Medicare eligibility threshold by states dental benefits in Medicaid.

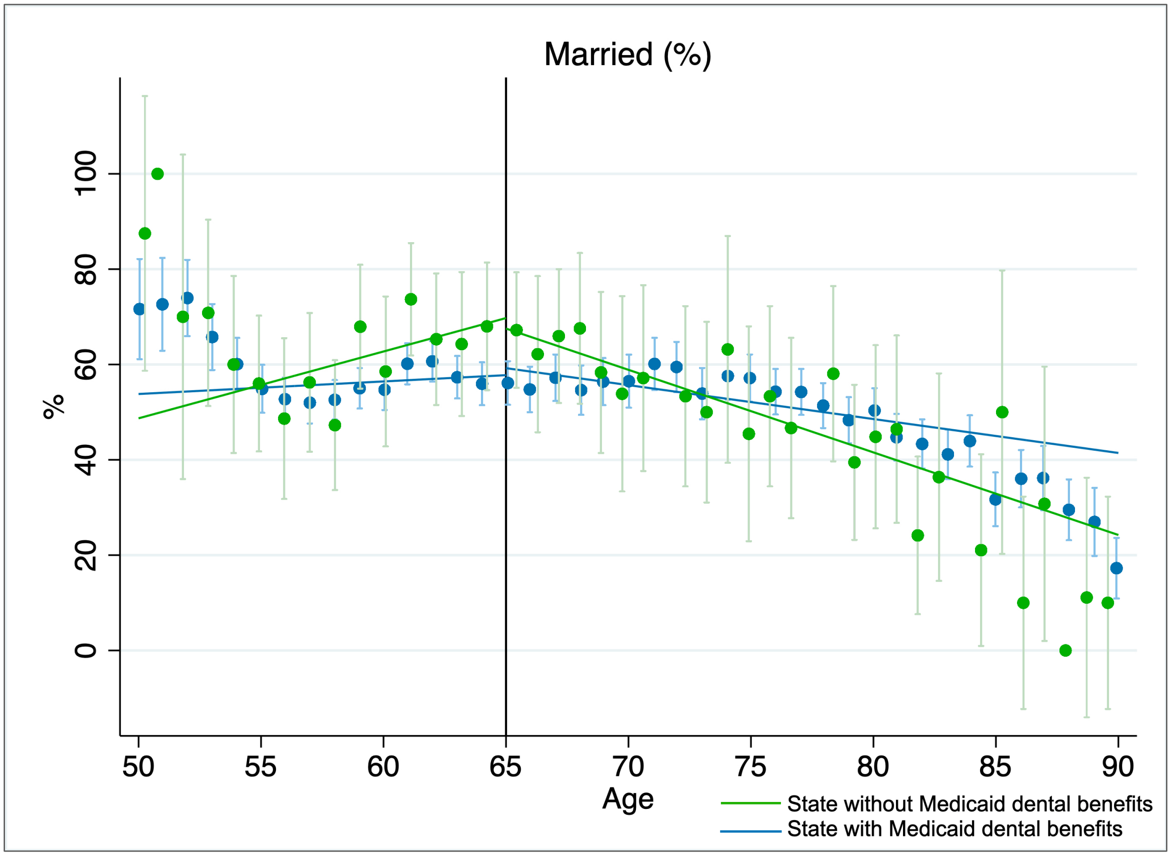

Note. Scatter plots of unadjusted proportion of married status above and below the Medicare eligibility threshold. Data are from the Health and Retirement Study (HRS) survey years 2014 to 2020. Study sample limited to adults ages 50 to 90 years with up to 12 years of education in ACA expansion states (28 states). All analyses are weighted by HRS survey weights and used robust standard errors clustered by individual.

© 2024 Elani HW et al. JAMA Health Forum.

eFigure 3. Sensitivity of regression discontinuity estimates to alternative bandwidth and kernel for medical coverage outcomes

Uniform kernel	Triangular kernel	Uniform kernel	Triangular kernel

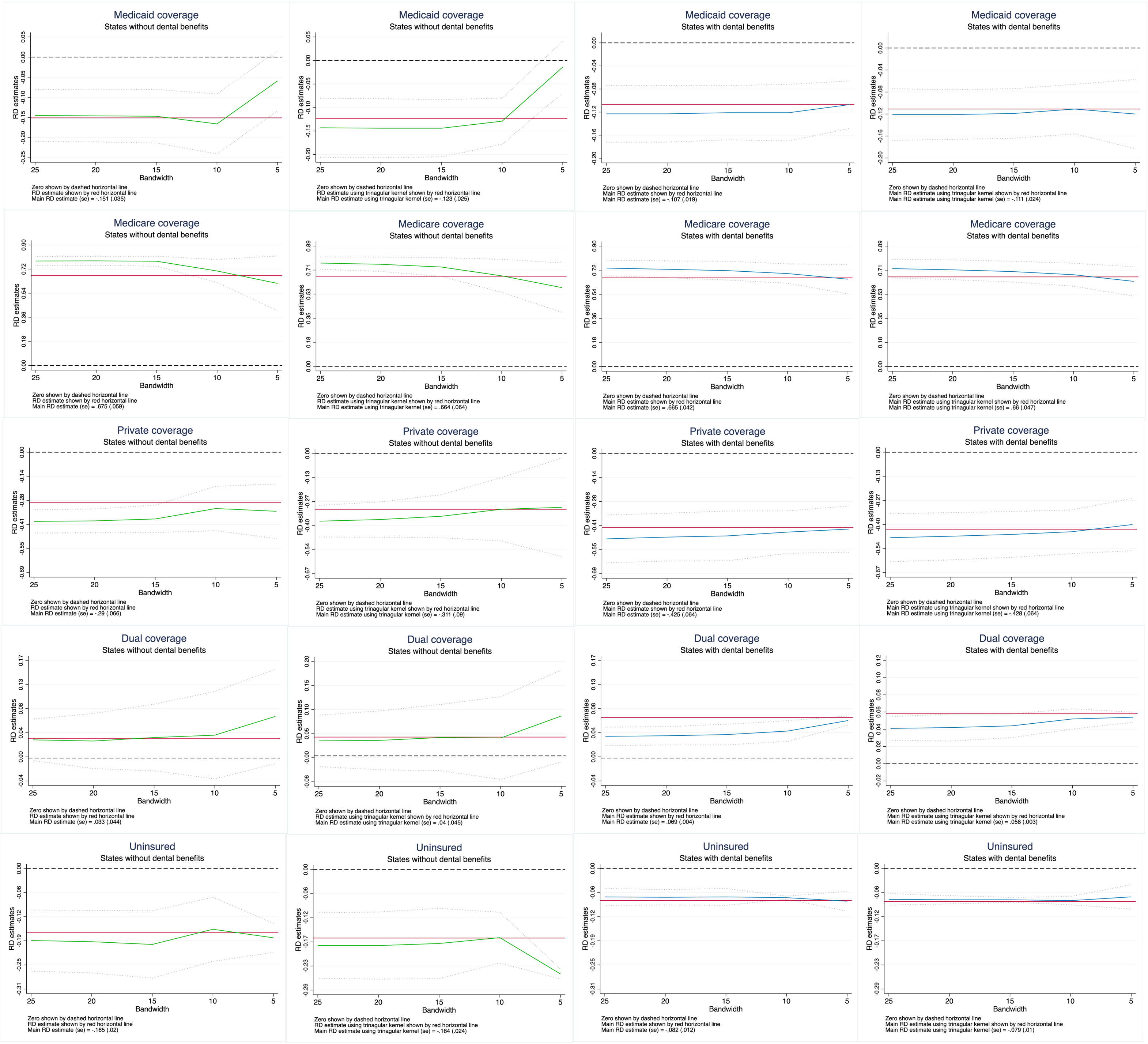

Note. Regression discontinuity estimate with different bandwidths around age 65. Adjusted discontinuities are estimated using local linear regression with a uniform kernel. Models included individual-level covariates, state fixed effects, and year fixed effects and used robust standard errors clustered by individual. Estimates using uniform kernel are weighted by HRS survey weights. Estimates using triangular kernel assigns more weight to individuals close to the threshold.

© 2024 Elani HW et al. JAMA Health Forum.

eFigure 4. Sensitivity of regression discontinuity estimates to alternative bandwidth and kernel for dental coverage, access, and out-of-pocket spending

Uniform kernel	Triangular kernel	Uniform kernel	Triangular kernel

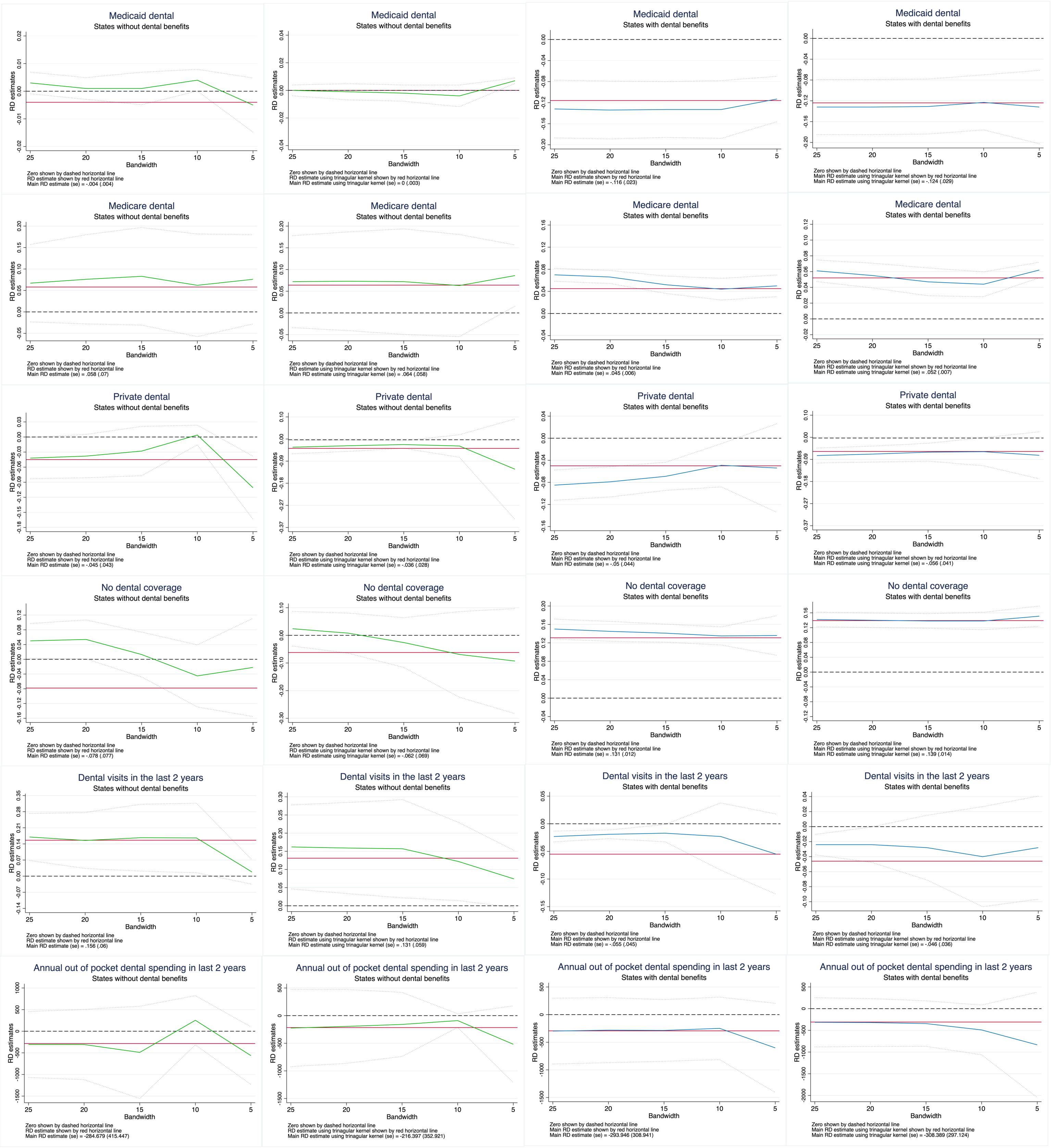

Note. Regression discontinuity estimate with different bandwidths around age 65. Adjusted discontinuities are estimated using local linear regression with a uniform kernel. Models included individual-level covariates, state fixed effects, and year fixed effects and used robust standard errors clustered by individual. Estimates using uniform kernel are weighted by HRS survey weights. Estimates using triangular kernel assigns more weight to individuals close to the threshold.

© 2024 Elani HW et al. JAMA Health Forum.
