# Supplementary material for: Dental Coverage and Care When Transitioning From Medicaid to Medicare
Source: JAMA Health Forum. 2024 Nov 22;5(11):e244165. doi: 10.1001/jamahealthforum.2024.4165 (PMC11584926; doi:10.1001/jamahealthforum.2024.4165)
Supplement: Supplement 2. — Data sharing statement [file jamahealthforum-e244165-s002.pdf]

Data Sharing Statement

Elani. Dental Coverage and Care When Transitioning from Medicaid to Medicare. *JAMA Health Forum*. Published November 22, 2024. doi:10.1001/jamahealthforum.2024.4165

Data
Data available: No

Additional Information
Explanation for why data not available: We accessed restricted data from the Health and Retirement study and per our Data Use agreement we can not share individual data
